# Adenosine A_3_ Receptor: From Molecular Signaling to Therapeutic Strategies for Heart Diseases

**DOI:** 10.3390/ijms25115763

**Published:** 2024-05-25

**Authors:** Ratchanee Duangrat, Warisara Parichatikanond, Wisinee Chanmahasathien, Supachoke Mangmool

**Affiliations:** 1 Department of Pharmacology, Faculty of Science, Mahidol University, Bangkok 10400, Thailand; ratchanee.duan@gmail.com; 2 Department of Pharmacology, Faculty of Pharmacy, Mahidol University, Bangkok 10400, Thailand; warisara.par@mahidol.ac.th; 3 Department of Pharmaceutical Sciences, Faculty of Pharmacy, Chiang Mai University, Chiang Mai 50200, Thailand; wisinee.c@cmu.ac.th; 4 Department of Pharmaceutical Care, Faculty of Pharmacy, Chiang Mai University, Chiang Mai 50200, Thailand

**Keywords:** adenosine, A_3_AR, A_3_AR agonist, heart diseases, heart failure, ischemia

## Abstract

Cardiovascular diseases (CVDs), particularly heart failure, are major contributors to early mortality globally. Heart failure poses a significant public health problem, with persistently poor long-term outcomes and an overall unsatisfactory prognosis for patients. Conventionally, treatments for heart failure have focused on lowering blood pressure; however, the development of more potent therapies targeting hemodynamic parameters presents challenges, including tolerability and safety risks, which could potentially restrict their clinical effectiveness. Adenosine has emerged as a key mediator in CVDs, acting as a retaliatory metabolite produced during cellular stress via ATP metabolism, and works as a signaling molecule regulating various physiological processes. Adenosine functions by interacting with different adenosine receptor (AR) subtypes expressed in cardiac cells, including A_1_AR, A_2A_AR, A_2B_AR, and A_3_AR. In addition to A_1_AR, A_3_AR has a multifaceted role in the cardiovascular system, since its activation contributes to reducing the damage to the heart in various pathological states, particularly ischemic heart disease, heart failure, and hypertension, although its role is not as well documented compared to other AR subtypes. Research on A_3_AR signaling has focused on identifying the intricate molecular mechanisms involved in CVDs through various pathways, including G_i_ or G_q_ protein-dependent signaling, ATP-sensitive potassium channels, MAPKs, and G protein-independent signaling. Several A_3_AR-specific agonists, such as piclidenoson and namodenoson, exert cardioprotective impacts during ischemia in the diverse animal models of heart disease. Thus, modulating A_3_ARs serves as a potential therapeutic approach, fueling considerable interest in developing compounds that target A_3_ARs as potential treatments for heart diseases.

## 1. Introduction

Adenosine initiates a wide range of cardiovascular effects upon binding to various subtypes of adenosine receptors (ARs), including A_1_AR, A_2A_AR, A_2B_AR, and A_3_AR, expressed within cells of the cardiovascular system [1,2]. During myocardial ischemic conditions, adenosine is released in large amounts and subsequently activated by A_1_AR and A_3_AR to confer ischemic preconditioning (IPC) in the heart [3,4]. Extensive research has focused on the role of A_1_AR in protecting the heart from damage, revealing that the stimulation of A_1_ARs can trigger protective responses within cardiac tissues [5,6]. However, recent studies have continuously revealed that A_3_AR also induces protective responses similar to those activated by A_1_AR [7,8]. However, the role of A_3_ARs is not as extensively documented in pathological cardiac tissues as other ARs. This is primarily due to the low expression of A_3_AR in the heart and disparities in its adenosine binding affinity between human and rodent models [9].

The A_3_AR subtypes are distributed throughout various tissues and organs such as the testes, lungs, kidneys, heart, brain, and spleen, although the level of expression varies among species [10]. High intensities of A_3_AR localization have been observed in several immune and inflammatory cells, including neutrophils, eosinophils, and mast cells, emphasizing the pivotal role of A_3_ARs in inflammation processes [11,12]. Interestingly, the differential expression of A_3_AR has been documented in both pathological and normal cells. The overexpression of A_3_ARs has been detected in several types of cancer cells, including leukemia, lymphoma, astrocytoma, melanoma, lung, breast, and renal carcinomas, whereas it is minimally expressed in normal cells [10,13].

Human A_3_AR was initially cloned and characterized in 1993 [14]. In terms of sequence similarity, A_3_AR structure presents a significant difference in species homolog. Human A_3_ARs are more closely related to those in sheep than in rats, exhibiting 85% versus 74% similarity, respectively [15], and equine A_3_ARs also show high similarity to both humans and sheep, suggesting unique ligand recognition and pharmacological behaviors, which pose challenges for fully understanding and characterizing these receptors [16]. The A_3_AR gene (*ADORA3*), a single-copy gene located on human chromosome 1p21–p13, features a coding region consisting of two exons separated by a single intron of approximately 2.2 kb, coding for 319 amino acids [17]. The promoter of the A_3_AR gene lacks a TATA motif but contains a CCAAT motif and binding sites for multiple transcription factors, such as specificity protein 1 (SP1), transcription factor nuclear factor interleukin 6 (NF-IL6), GATA1, and GATA3, suggesting its significant role in immune responses [15].

A_3_AR plays a complex role in the cardiovascular system, as its activation helps to limit the injury processes occurring in the heart during ischemia [4,16]. Additionally, during the reperfusion phase, A_3_AR exerts an anti-inflammatory action, thereby minimizing further damage to the heart tissue [18]. Studies conducted on both cardiomyocytes and intact hearts of rats subjected to ischemia/reperfusion (I/R) showed that A_3_AR agonists can reduce the infarct size associated with the upregulation of pro-survival signaling pathways such as extracellular signal-regulated kinase 1/2 (ERK1/2) and phosphoinositide 3-kinase (PI3K)/Akt pathways [19,20]. Moreover, the stimulation of A_3_ARs protects against cardiotoxic side effects induced via chemotherapy, indicating the cardioprotective effects of A_3_AR signaling [21,22]. The activation of A_3_ARs has been shown to influence vascular tone in multiple ways, including the inhibition of coronary flow in isolated mouse aorta hearts [23], the induction of vasoconstriction in hamster arterioles [24], and the restoration of vascular reactivity following hemorrhagic shock in rats [25].

A balance of A_3_AR expression levels is essential for optimal cardiac function. For instance, the overexpression of A_3_ARs can lead to decreased heart rate, the preservation of energetics, and the protection of the heart against ischemic damage [26]. However, higher levels of A_3_AR expression are associated with the development of dilated cardiomyopathy [27]. Remarkably, A_3_AR signaling is involved in growth promotion in the heart, as the genetic ablation of A_3_AR attenuated pathological cardiac remodeling characterized by hypertrophic growth and fibrotic changes [28]. While a direct cardioprotective effect mediated by A_3_AR has been observed, the A_3_AR expression level in cardiomyocytes is relatively low. Therefore, there is a potential for indirect protection, possibly by affecting the function of immune cells such as mast cells and neutrophils, which abundantly express A_3_ARs. However, species differences need to be considered in AR signaling-mediated mast cell degranulation [11,12]. Overall, A_3_AR plays a complex role in the cardiovascular system, involving both direct and indirect mechanisms. Developing compounds targeting A_3_ARs, either agonists or antagonists, could potentially be manipulated to offer cardiovascular therapeutic benefits. However, it requires careful consideration due to the potential risk of adverse effects.

Building on these considerations, this review aims to elucidate the essential role of A_3_ARs in the heart. It offers an extensive review of the signal transduction pathways associated with A_3_ARs and explores the use of A_3_AR agonists and antagonists in various models of pathological heart conditions.

## 2. The Distribution of A_3_ARs in the Cardiovascular System

The cardiovascular system is composed of various cell types, including cardiomyocytes, cardiac fibroblasts, smooth muscle cells, and endothelial cells. Although A_3_AR is expressed in the atria and left ventricle of rat hearts, its mRNA levels are relatively low compared to those in other tissues (e.g., testes, kidneys, lungs, brain, liver, eyes, and spleen) (reviewed in [15]), and this expression is downregulated during hypertension [29]. There are also significant differences in expression levels within and between species. For instance, high levels of A_3_AR mRNA are reported in the testes and mast cells of rats, while other rat tissues, including the cardiovascular system, exhibit low levels of this mRNA [10,30]. In humans, the lungs and liver express high amounts of A_3_AR mRNA, whereas low levels have been found in the aorta and brain [14].

Additionally, A_3_AR expression has been identified in blood vessels, such as rat thoracic aorta [29] and human coronary and carotid arteries [31,32]. In situ hybridization results detected A_3_AR mRNA expression in the vascular smooth muscle layer and, to a lesser extent, in the endothelial layer of the mouse aorta [33]. Interestingly, a recent study has demonstrated that A_1_AR mRNA is the most abundant, followed by A_2_AR and A_3_AR in human right atrial tissue [34]. In addition, the stimulation of A_3_ARs regulated spontaneous calcium release in isolated human atrial myocytes [34], indicating the role of A_3_AR in calcium homeostasis. The stimulation of A_3_AR, using its selective agonist, reduced infarction size in isolated rabbit hearts, exerting a cardioprotective effect in I/R injury [35]. These findings suggest that, despite the limited data on its distribution in cardiovascular tissues, A_3_AR plays a crucial role in cardiovascular protective effects.

## 3. A_3_AR Signaling in the Cardiovascular System

### 3.1. G_i_ Protein-Dependent Signaling

**Figure 1:**
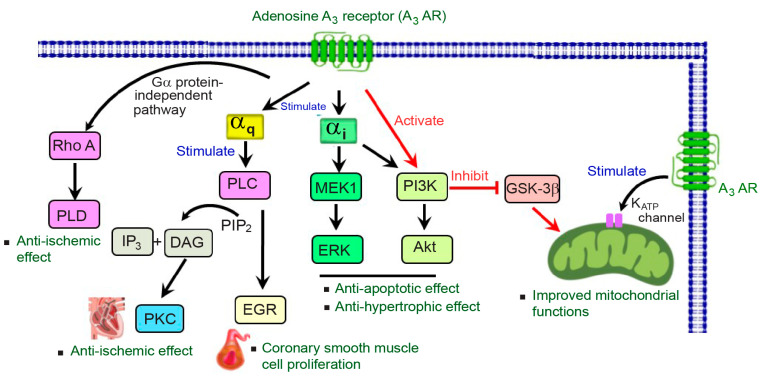
A_3_AR-mediated signaling pathways for cardiovascular protective effects. The signal transduction pathway of A_3_ARs in the cardiovascular system involves the activation of G_αq_ and G_αi_ proteins. When A_3_AR is coupled with G_αq_, it stimulates PLC activity and induces transcription factors EGR, leading to the increased proliferation of coronary smooth muscle cells. Additionally, the coupling of A_3_AR with G_αq_ leads to elevated levels of IP_3_ and DAG, with the latter activating PKC, which mediates an anti-ischemic effect in isolated hearts. A_3_AR can also transduce its signals independently of G_α_ protein mediating RhoA-PLD interaction, thereby protecting the heart from ischemic damage in cardiomyocytes. When coupled with the G_αi_ protein, A_3_AR inhibits GSK-3β activity through the PI3K pathway, improving mitochondrial function. In addition, A_3_AR activates mitoK_ATP_, further enhancing mitochondrial function. Furthermore, A_3_AR exhibits an anti-apoptotic effect by activating the PI3K/Akt pathway, and an anti-hypertrophic effect through the G_αi_-dependent activation of MEK1/ERK, ultimately conferring cardioprotective effects in cardiomyocytes. Abbreviations: DAG, diacylglycerol; EGR, growth response factors; ERK, extracellular signal-regulated kinase; GSK-3β, glycogen synthase kinase-3 beta; IP3, inositol triphosphate; MEK1, mitogen-activated protein kinase kinase 1; mitoKATP, mitochondrial ATP-sensitive potassium channel; PKC, protein kinase C; PI3K, phosphoinositide 3-kinase; PLC, phospholipase C; PLD, phospholipase D; RhoA, Ras homolog family member A.

A_3_AR is a cell surface receptor belonging to the family of G protein-coupled receptors (GPCRs). Upon binding to its agonist, A_3_AR can trigger both G protein-dependent and independent signaling pathways, which vary by cell type; these pathways are associated with anticancer, anti-inflammatory, and cardioprotective effects (reviewed in [36,37]). A_3_AR signaling is characterized by its interaction with both G_i_ and G_q_ proteins. This dual coupling enables A_3_AR to mediate a diverse range of biological responses depending on the type of G protein activated (reviewed in [36,37]). Notably, A_3_AR demonstrates a significant association with the G_i_ protein, resulting in the inhibition of cyclic adenosine monophosphate (cAMP) production and thereby activating the mitogen-activated protein kinase (MAPK) pathway, including ERK1/2 and p38 [38] (Figure 1).

A_3_AR, when coupled with G_i_, results in the inhibition of adenylyl cyclase (AC), leading to a decrease in cAMP formation. The low levels of cAMP subsequently decrease protein kinase A (PKA) activity, which in turn attenuates the phosphorylation of target proteins and eventually affects a series of intracellular events [13]. Following A_3_AR coupling with G_i_ protein, the inhibition of cAMP formation indirectly reduces glycogen synthase kinase-3 beta (GSK-3β) activity, leading to an increase in the phosphorylation of β-catenin. This prevents β-catenin from translocating to the nucleus, resulting in the decreased expression of various target genes that regulate cell cycle progression, such as cyclin D1 and c-myc, ultimately inhibiting cell growth (reviewed in [13,15,37]). Of note, the activation of G_i_ protein-dependent signaling can have detrimental effects on the heart. Previous studies have demonstrated that the cardiac-specific expression of a G_i_-coupled receptor, a synthetic receptor designed to interact with the G_i_ protein, led to adverse outcomes in mouse hearts, including a slowing heart rate, arrhythmia, and lethal cardiomyopathy [39,40].

The PI3K/Akt and ERK signaling cascades are recognized for their pivotal roles in cell survival and cardiac defensive mechanisms, with A_3_AR capable of transducing its signal through the activation of these pathways [19,20]. In rat cardiomyocytes, the stimulation of A_3_ARs leads to the phosphorylation of Akt through the G_i_/G_o_ protein. Simultaneously, the inhibition of PI3K prevented the activation of Akt, indicating the role of PI3K as a downstream mediator of the G_i_ protein-dependent signaling pathway for Akt activation [41]. In addition, the role of A_3_ARs in cardioprotection during IPC has been documented, as the same group of investigators demonstrated the preconditioning effect of adenosine involved with A_1_AR- and A_3_AR-mediated anti-apoptotic effects via the mitogen-activated protein kinase kinase 1 (MEK1) and ERK1/2 pathway [20]. Interestingly, A_3_AR-induced IPC also involved a PI3K-dependent pathway in rat cardiomyocytes [20]. Moreover, previous studies have also reported the pharmacological preconditioning effects of resveratrol, a compound found abundantly in grapes, in protecting the heart, mainly mediated by A_3_ARs [42,43]. The stimulation of A_3_ARs leads to the activation of two different signaling pathways, including the PI3K/Akt pathway and the response element-binding protein (CREB)-dependent pathway, through both Akt-dependent and -independent signaling in the heart tissues. Both pathways lead to the activation of apoptosis regulator Bcl-2 signaling, providing survival signals to the heart [42,43].

Furthermore, A_3_AR signaling mediated the activity of MAPK signaling pathways, such as ERK1/2, which has been documented in various cell models [15]. For instance, the triggering of A_3_ARs in Chinese hamster ovary (CHO) cells expressing human A_3_ARs resulted in the robust activation of ERK1/2 through G_i/o_ protein-dependent signaling pathway, involving the activation of PI3K, Ras, and MEK to induce ERK1/2 activation [44,45]. Moreover, in the rat heart, the triggering phase of beta-adrenergic during IPC led to the generation of adenosine and reactive oxygen species (ROS), which then stimulated A_3_ARs, resulting in the activation of specific signaling pathways, including PI3K/Akt and ERK. This may lead to the inhibition of GSK-3β activity, ultimately closing the mitochondrial permeability transition pore (mPTP) and providing cardioprotection [46]. In addition, A_3_AR-mediated cardioprotection involved the activation of pro-survival signaling pathways including MEK1/2-ERK1/2 and PI3K/Akt (Figure 1), resulting in decreased caspase-3 activity, a marker of cellular apoptosis, and protecting the rat hearts from I/R injury [19].

The role of p38 MAPK has also been demonstrated in rat cardiomyocytes. The activation of p38 MAPK occurred downstream from the opening of the mitochondrial ATP-sensitive potassium (mitoK_ATP_) channels following A_3_AR activation, thereby participating in the intracellular signaling pathway and contributing to a protective effect on the heart against ischemic injury in rats [47]. While the precise mechanism of p38 MAPK activation remained unclear, it is suggested that ROS generated upon mitoK_ATP_ channel activation may trigger this process [47]. However, in certain cell types, such as A375 human melanoma cells, A_3_AR stimulation did not promote ERK1/2 or p38 MAPK activation. Instead, it transduced its signal through the PI3K/Akt signaling pathway, resulting in a reduction in the basal levels of ERK1/2 phosphorylation and the inhibition of cell proliferation [48]. Furthermore, in response to A_3_AR stimulation, the inhibition of both p38 MAPK and nuclear factor kappa B (NFκB) pathways has been observed, associated with the suppression of inflammatory processes in human synoviocytes derived from osteoarthritis patients [49].

In the heart, several downstream mediators were reported to be involved in the cardioprotective effects of A_3_ARs, including GSK-3β, mitoKATP channel, protein kinase C (PKC), ROS, connexin 43, and mPTP (reviewed in [13]). The stimulation of A_3_ARs suppresses GSK-3β activity through the PI3K/Akt pathway in isolated rat hearts, leading to the inhibition of mPTP. This, in turn, protects the heart from I/R injury by reducing infarction size [50].

### 3.2. G_q_ Protein-Dependent Signaling

A_3_AR interacts with G_q_ to activate phospholipase C (PLC), which then produces inositol triphosphate (IP_3_), increasing intracellular Ca^2+^ levels, thereby regulating various cellular activities and activating PKC [10,13,51] (Figure 1). Although the upstream signaling pathways of PKC are not fully understood, previous studies have underscored the essential role of A_3_AR and PKC in the heart. For example, the activation of A_3_ARs has been shown to induce delayed preconditioning cardioprotection in the mouse heart, specifically through the activation of PKC-δ isoform via mechanisms yet to be fully elucidated [52]. It has been suggested that A_3_AR-mediated PKC activation may trigger various downstream mediators, including ERK1/2 and p38 MAPK, and contribute to cardioprotective effects [2,15]. Moreover, the modulation of Ca^2+^ release from the cardiac sarcoplasmic reticulum has been implicated in PKC activation, independent of PLC activation through A_3_ARs in isolated rat hearts [53].

IPC has been observed to protect guinea pig hearts by triggering a signaling cascade involving A_3_AR stimulation, and the subsequent activation of PKC-ε and mitochondrial aldehyde dehydrogenase type-2 (ALDH2) in cardiac mast cells [54]. This series of reactions helps to prevent the detrimental effects associated with the renin–angiotensin system (RAS) activation during I/R injury [54]. In the rat brain, the oxygen/glucose deprivation-induced depression of synaptic transmission is mediated by the coactivation of metabotropic glutamate receptor 1 (mGluR1) and A_3_AR [55]. Interestingly, the blocking of A_3_AR and PKC, along with a decrease in internal Ca^2+^, has been shown to prevent this depression of synaptic transmission in CA3 pyramidal neurons, underscoring the significant role of PLC-dependent G protein signaling in ischemic conditions [55].

### 3.3. ATP-Sensitive Potassium Channels (K_ATP_)

Additionally, A_3_AR stimulation facilitated the activation of the ATP-sensitive potassium (K_ATP_) channels, leading to their opening and conferring cardioprotective effects during I/R injury in rabbit hearts [56]. However, it remained uncertain whether the channel opens as a direct result of A_3_AR stimulation or following the activation of PKC [56]. Furthermore, the function of A_3_ARs is also involved in the activation of the sarcolemmal K_ATP_ channels, offering protection against reperfusion injury in mouse hearts [57]. However, the molecular mechanism underlying this effect is not elucidated in the study.

### 3.4. G Protein-Independent Signaling

Interestingly, A_3_AR signaling has been shown to mediate both G protein-dependent and -independent pathways (Figure 1). The stimulation of A_3_AR can transduce its signals independently of traditional G protein-mediated signaling pathways, such as phospholipase D (PLD) and Ras homolog family member A (RhoA) [13]. In cultured chick embryo cardiomyocytes, the RhoA-PLD interaction is a key player in A_3_AR signaling, protecting heart cells from ischemic injury [58]. However, the underlying mechanisms of cardioprotective effects during cardiac injury mediated by G protein-independent pathways are not quite understood.

## 4. The Role of A_3_ARs in Heart Diseases

Over time, as our understanding of the modulation of A_3_ARs has advanced, its pivotal roles in cardiac physiology and pathology have become increasingly apparent. This has led to a focused effort to explore the potential of selective A_3_AR agonists for protecting the heart. Various A_3_AR-specific agonists, including IB-MECA, Cl-IB-MECA, CP-532903, and CP-608039, have been examined for their effectiveness in treating a range of heart conditions, such as I/R injury and cardiac remodeling [5,15,37]. The cardioprotective effects of A_3_AR agonists have been studied across different animal models, including rodents and rabbits, as shown in Table 1. Notably, variations in agonist profiles for treatment efficacy have been observed in accordance with interspecies differences [59]. Furthermore, some A_3_AR agonists may interact with other subtypes of ARs under different conditions, emphasizing the need for the cautious interpretation of results [59].

### 4.1. Contributions of A_3_ARs in Ischemic Heart Disease

The activation of A_3_ARs has shown beneficial outcomes in cardiac ischemia in both in vitro and in vivo studies with various agonists targeting A_3_AR reported for cardiac ischemic protection [57,60,61,62,63]. In 1994, a study revealed that treatment with aminophenylethyladenosine (an A3AR agonist) produced protective effects similar to adenosine against I/R injury. The blocking of A_3_AR with BWA1433, an A_3_AR antagonist, abolished this protective effect in rabbit hearts, with A_1_AR not mediating this outcome [64]. In mice models of I/R injury, a reduction in infarct size was observed following A_3_AR activation using Cl-IB-MECA (A_3_AR agonist). However, this beneficial effect was abolished in A_3_AR-knockout mice, confirming the essential role of A_3_ARs in ischemic heart protection [65] (Table 1).

Moreover, the modulation of A_3_AR levels in the heart is crucial for optimal cardiac function and protection against ischemia [27]. By employing cardiomyocyte-specific A_3_AR transgenic mice engineered to express A_3_ARs at varying levels, including low (one copy of the transgene) and high (six copies of the transgene), the data revealed that while mice with low levels of A_3_ARs exhibited no discernible cardiac abnormalities, those with high levels experienced several cardiac issues [27]. Nonetheless, both sets of transgenic mice demonstrated improved outcomes in reducing infarct size compared to wild-type mice, indicating a conferred protection against I/R injury.

The agonism of A_3_AR consistently led to a reduction in infarct size across various study models of I/R injury (Table 1). The efficacy of CP-532903 (piclidenoson; A_3_AR agonist) in protecting against I/R injury has been demonstrated in mice [60]. In a rabbit model of regional ischemia, CP-532903 effectively decreased infarct size without inducing hemodynamic liabilities [61]. Additionally, CP-532903 showcased its ability to reduce infarct size in an in vivo mouse model of infarction and to enhance functional recovery in isolated mouse hearts subjected to I/R injury. These protective effects were attributed to the activation of sarcolemmal K_ATP_ channels [57], and similar cardioprotective mechanisms were also observed in adult mouse ventricular cardiomyocytes [60].

The administration of IB-MECA has shown significant promise in reducing infarct size by 61% compared to the control group in conscious rabbits, providing the cardioprotective effects mediated through the PKC-dependent pathway against I/R injury without causing significant hemodynamic changes [62]. In agreement with another study, preconditioning with IB-MECA prior to low-flow ischemia in Langendorff rat hearts improved the recovery of heart function and reduced apoptosis after 150 min of reperfusion, indicating that the activation of A_3_AR signaling can reduce cell death following I/R injury [66]. Moreover, CP-608039, an A_3_AR agonist, with high water solubility suitable for intravenous route administration, has been developed for preventing perioperative myocardial ischemic injury [61,67]. Currently, there is controversy regarding the timing of cardioprotective effects of A_3_AR signaling, with ongoing debate on whether pre-ischemic or post-ischemic treatment with A_3_AR agonists is more beneficial [7,15], indicating the need for further research.

**Table 1:** Protective effects of A_3_AR agonists in preclinical models of heart diseases.

Agents	Models	Main Findings
**Ischemia and myocardial infarction**		
CP-532903 [57]	Mice model of ischemia-reperfusion (I/R) injury	▪ Reduced infarct size and improved contractile function ▪ Activated sarcolemma K_ATP_ channels
CP-532903 [60]	Isolated rat hearts and isolated cardiomyocytes	▪ Protected the heart depended on the A_3_AR expression level
CP-532903 and CP-608039 [61]	Rabbit model of I/R injury	▪ Reduced infarct size without hemodynamic changes
Namodenoson (2Cl-IB-MECA; CF-102) [65]	Mice model of I/R injury	▪ Reduced infarct size by stimulating A_3_ARs
Namodenoson (2Cl-IB-MECA; CF-102) [19]	Isolated rat hearts with I/R injury	▪ Reduced infarct size and necrosis ▪ Reduced apoptosis and caspase-3 activity ▪ Exhibited cardioprotection via MEK1/2-ERK1/2 and PI3K-Akt pathways
Namodenoson (2Cl-IB-MECA; CF-102) [18]	Mice model of I/R injury	▪ Reduced infarct size ▪ Reduced leukocyte infiltration into the myocardium ▪ Activated pro-survival signaling and reduced inflammation
Piclidenoson (IB-MECA; CF-101) [62]	Rabbit model of I/R injury	▪ Protected against both reversible (stunning) and irreversible (infarction) injuries via PKC pathway without hemodynamic changes
Piclidenoson (IB-MECA; CF-101) [4]	Isolated human atrial muscle	▪ Improved contractile function against ischemic preconditioning
Piclidenoson (IB-MECA; CF-101) [68]	Rabbit model of I/R injury	▪ Attenuated myocardial stunning ▪ Provided cardioprotection by stimulating A_3_ARs
**Heart failure**		
N^6^-cyclopentyl-adenosine (CPA; full agonist) [28]	Wild-type and A_3_AR-knockout mice with transverse aortic constriction-induced pressure overload	▪ Attenuated cardiac hypertrophy, fibrosis, and myocardial dysfunction ▪ Prevented heart failure

#### 4.1.1. Anti-Inflammatory Effects

I/R injury poses a threat to heart tissue by triggering inflammation and oxidative stress [69]. However, treatment with Cl-IB-MECA (A_3_AR agonist) during reperfusion has demonstrated the ability to reduce infarct size by decreasing leukocyte recruitment. This reduction is attributed to the inhibition of neutrophil migration and pro-inflammatory actions through the activation of A_3_ARs in bone marrow-derived cells, effectively suppressing the inflammatory response during reperfusion injury in a mouse model [18]. Furthermore, the activation of A_3_ARs with 2-CL-IB-MECA (A_3_AR agonist) led to the activation of pro-survival signaling pathways, including the MEK1/2-ERK1/2 and PI3K/Akt pathways. This activation was accompanied by a decrease in apoptosis, necrosis, and caspase 3 activity in rat cardiomyocytes in response to hypoxia/reoxygenation injury [19].

The benefits of stimulating A_3_AR signaling in preconditioning the heart have been well documented in several studies [20,64,66]. However, paradoxical effects of A_3_AR were observed in multiple experiments. One study revealed that while A_3_AR activation is typically beneficial in protecting the heart from I/R injury under normal circumstances, A_3_AR-knockout mice surprisingly displayed a post-ischemic recovery response compared to wild-type mice [70]. Consistently, mice lacking A_3_AR exhibited smaller infarct sizes compared to wild-type mice [71]. Moreover, A_3_AR exacerbated I/R injury, possibly through involvement in promoting inflammatory responses, and it is not essential for the protective mechanism of IPC [71]. Indeed, the paradoxical effect of A_3_AR might be due to the species differences. A_3_AR signaling in mast cells, particularly in rodents, may trigger a pro-inflammatory response through mast cell degranulation, potentially harming the heart [11,12,54]. Consequently, mice lacking A_3_AR may exhibit beneficial effects on cardiac function. This evidence highlighted the complexity of A_3_AR signaling mechanisms and suggested a potential role of inflammation in both I/R injury and IPC [59,72].

#### 4.1.2. Protection of Oxidative Stress, Apoptosis, and Mitochondrial Dysfunction

The stimulation of A_3_ARs has been found to exhibit a cardioprotective effect against doxorubicin-induced cardiotoxicity. In cultured rat cardiomyocytes, treatment with Cl-IB-MECA (A_3_AR agonist) effectively mitigated the adverse effects of doxorubicin through multiple mechanisms [73]. Cl-IB-MECA exerted its cardioprotective effects by inhibiting cardiac cell damage, which was mediated by the activation of cellular antioxidant enzymes, leading to a reduction in free-radical generation and lipid peroxidation [73]. Furthermore, Cl-IB-MECA demonstrated the ability to protect cells from mitochondrial damage, thereby preventing a decrease in ATP levels [73]. Consistent with these findings, pretreatment with Cl-IB-MECA was shown to attenuate left ventricular dysfunction in rat hearts, improve SR calcium storage capacity, and prevent mitochondrial calcium overload, thus protecting the cardiomyocytes [22]. Additionally, the activation of A_3_ARs with Cl-IB-MECA was associated with the activation of pro-survival signaling pathways, leading to a reduction in caspase-3 activity in rat hearts, thereby conferring cardioprotection against I/R injury [19].

### 4.2. Impacts of A_3_ARs on Heart Failure

Endogenous adenosine can protect the overloaded heart against the development of pathological heart failure [74]. A study showed that mice deficient in extracellular adenosine production, stemming from a deletion in the CD73 gene, showed maladaptive tissue remodeling with the induction of cardiomyocyte hypertrophy, fibrosis, and left ventricular dilation and dysfunction in contrast to their wild-type counterparts under chronic systolic overload [75]. However, the role of specific AR subtypes that contribute to this pathological heart has rarely been discussed. It has been noted that alterations in A_3_AR levels exert a significant influence on both cardiac phenotypes and functions. The cardiomyocyte-specific overexpression of A_3_ARs in mice led to dilated cardiomyopathy with an elevation of hypertrophic markers and systolic dysfunction, whereas a low level of A_3_ARs showed no detectable deleterious events [27]. Remarkedly, mice harboring more than six copies of the A_3_AR transgene experienced even worse outcomes, succumbing within 4 weeks of birth [27]. Consistently, A_3_AR overexpression in the heart resulted in dose-dependent atrioventricular (AV) block and sinus nodal dysfunction, as well as the induction of bradycardiomyopathy in mice [76]. These observations collectively underscore the deleterious impact of amplified A_3_AR signaling on cardiac functions, especially in the failing heart. Manipulating A_3_AR levels in the heart could offer possibilities for heart failure patients, and A_3_ARs could be considered a potential candidate for gene therapy in the context of heart failure.

#### 4.2.1. Hypertrophic Effects

Pressure overload is a critical factor in cardiovascular diseases, particularly hypertension. The A_3_ARs have been shown to have detrimental effects in animal models of pressure overload as shown in Table 2. Following chronic pressure overload, A_3_AR-knockout mice exhibited a decrease in left ventricular hypertrophy and dysfunction, along with a reduction in myocyte size and fibrosis compared to wild-type mice [28]. The antagonism of A_3_ARs with MRS1911 not only reduced oxidative stress and the elevated levels of hypertrophic markers but also potentiated the anti-hypertrophic effects of CADO, an adenosine analog, in phenylephrine-induced hypertrophy in isolated mouse cardiomyocytes [28]. Additionally, MAPKs, ERK, and JNK, which are often activated and contribute to cardiac hypertrophy and heart failure, were also reduced in the presence of an A_3_AR antagonist [28]. These findings collectively suggest that A_3_AR plays a significant role in mediating adverse effects in the pressure-overloaded heart.

#### 4.2.2. Fibrotic Effects

There is a limited number of studies focusing exclusively on the role of A_3_ARs in cardiac fibrosis. However, a study by Lu and colleagues [28] shed light on this aspect by demonstrating that A_3_AR-knockout mice exhibited a significant reduction in left ventricular fibrosis compared to wild-type mice in a model of pressure overload (Table 2). Moreover, the previous findings showed that mice subjected to an early-life reduction in nephron numbers combined with chronic high salt intake (UNX-HS mice) developed hypertension and exhibited cardiac hypertrophy and fibrosis along with renal injury compared to controls [77]. Interestingly, A_3_AR-knockout mice displayed a significant attenuation of these pathologies and showed higher baseline levels of pro- and anti-inflammatory cytokines. This elevation in immune homeostasis may contribute to the resistance of mice lacking A_3_AR to UNX-HS-induced renal and cardiac pathologies [77]. In summary, these studies collectively suggested that A_3_AR signaling played a significant role in mediating the pathological effects of cardiovascular and renal injury.

**Table 2:** Detrimental effects of A_3_ARs in heart diseases.

Agents	Models	Main Findings
2-Chloroadenosine (adenosine analog) [28]	Wild-type and A_3_AR-knockout mice with transverse aortic constriction-induced pressure overload	▪ A_3_AR knockout had no effect on cardiac structure and functions in the normal heart ▪ A_3_AR knockout attenuated myocardial hypertrophy, fibrosis, and dysfunction
Cl-IB-MECA (2Cl-IB-MECA; CF-102) [78]	Wild-type and A_3_AR-knockout mice aorta	▪ Induced aorta contraction in wild-type mice and negligible effects in A_3_AR-knockout mice ▪ Mediated aorta contraction via COX-1 signaling ▪ Played a role in cardiovascular inflammation, including hypertension and atherosclerosis

#### 4.2.3. Effects on Cardiac Contractility and Heart Rate

The lack of hemodynamic effects of A_3_AR stimulation has been documented in several models. Previous findings revealed that the agonism of A_3_AR with IB-MECA did not produce any effects on heart rate or blood pressure measured for 1 h, even at higher doses of up to 300 μg/kg, unlike other AR agonists such as CCPA (A_1_AR agonist) and CGS 21680 (A_2A_AR agonist), which altered these hemodynamic parameters in the rabbit model [62]. In addition, IB-MECA showed no impact on ischemic contractility, as evidenced by the absence of any significant change in the contractile rate and force following I/R injury in preconditioned rat hearts [66]. In the rabbit model of I/R injury, CP-532903 (A_3_AR agonist) remarkably reduced infarct size in a dose-dependent manner without notably affecting hemodynamic variables such as mean arterial pressure, heart rate, and contractile rate and force, compared to the control group [61]. Moreover, following I/R, A_3_AR-overexpressing mice showed better preservation of cardiac function and ATP energy stores, leading to cardioprotection [26]. Overall, these suggested the protective role of A_3_AR in I/R injury without affecting the overall hemodynamic parameters. Nevertheless, it should be noted that mice overexpressing A_3_AR exhibited a lower basal heart rate and contractility compared to wild-type mice [26].

The role of A_3_ARs in arrhythmias is less well defined compared to other AR subtypes. However, an in vivo study has demonstrated that the overexpression of A_3_ARs in mice can considerably alter the electrophysiological function of the heart, particularly affecting the sinus and AV nodes [76]. Mice overexpressing A_3_ARs exhibited various pathological changes, including complete AV block, sinus bradycardia, atrial enlargement, and fibrosis [76]. Furthermore, elevated levels of A_3_AR expression were associated with the development of bradycardia–tachycardia syndrome, a type of cardiac arrhythmia, and may contribute to bradycardiomyopathy [76].

### 4.3. Involvement of A_3_ARs in Hypertension

A_3_AR has shown significant implications for vascular functions with extensive distributions in endothelial cells and vascular smooth muscle cells, reinforcing the idea that A_3_AR signaling may play a crucial role in hypertension and the pathophysiology of vascular diseases [78]. Previous studies have demonstrated that A_3_AR signaling is involved in vascular contraction [78,79] and that A_3_AR regulates cAMP levels and influences blood pressure in response to adenosine in the mice model [33]. The role of A_3_ARs on endothelium-dependent contraction was confirmed in mouse aorta [78]. Treatment with Cl-IBMECA, an A_3_AR agonist, induced contractions in wild-type mouse aorta with an intact endothelium but not in endothelium-denuded tissue, with this effect being abolished in A_3_AR-knockout mice (Table 2). Interestingly, A_3_AR-mediated contraction primarily relied on the endothelial cyclooxygenase-1 (COX-1) signaling pathway, contributing to vascular tone regulation [78]. Subsequent findings further corroborated the role of A_3_ARs in vascular contraction using the A_3_AR-knockout mice model, suggesting that vascular contraction involved ROS production mediated by A_3_ARs through the activation of nicotinamide adenine dinucleotide phosphate (NADPH) oxidase subtype 2 or NOX2 [79]. In conditions of hemorrhagic shock in rats, a decrease in A_3_AR expression was observed, correlating with diminished vasoreactivity to norepinephrine-induced vascular contraction response [25]. However, in the rat hemorrhagic shock model, exposure to IB-MECA considerably improved the response of the abdominal aorta to norepinephrine, potentially augmenting vasoconstriction and restoring the vascular function of vascular smooth muscle cells [25].

A_3_AR signaling was found to mediate the proliferation of vascular cells since the A_3_AR-induced proliferation of human coronary smooth muscle cells occurred through the activation of PLC and the induction of early growth response factors (EGRs), EGR2 and EGR3 [31]. In aortic vascular smooth muscle cells, A_3_AR-knockout mice exhibited decreased cell proliferation alongside an increase in the level of lysyl oxidase, an enzyme crucial for maintaining the structure and function of blood vessels through its involvement in crosslinking processes of the extracellular matrix [80]. However, A_3_AR deficiency alone is not significant enough to control the vascular response to injury or protect against atherogenesis in vivo [80]. In addition, A_3_AR is involved in regulating vascular functions in nonvascular cell types, such as mast cells. A_3_AR induced arteriolar constriction through mast cell degranulation in hamster cheek pouch arterioles, thus mediating vasoconstriction [24].

## 5. The Role of A_3_AR Agonists in the Treatment of Heart Diseases

Although the findings from animal models of heart disease in various previous studies remain inconclusive, earlier research has shown that several A_3_AR agonists, including piclidenoson (IB-MECA) and namodenoson (2Cl-IB-MECA), effectively shield hearts from I/R injury in various animal studies (Table 1). However, their practical application in clinical settings, particularly for patients with acute myocardial infarction, is restricted as they need to be administered before the onset of ischemia. Recent research suggests that the cardioprotective impact of preconditioning manifests early in reperfusion, primarily by targeting the mPTP opening via the activation of reperfusion injury salvage kinases (RISKs) like ERK and PI3K [81,82]. Similarly, past studies have shown that namodenoson and piclidenoson offer cardioprotection against I/R injury when administered upon reperfusion in animal models (Table 1). Thus, an A_3_AR agonist could potentially be suitable for patients with acute myocardial infarction; however, its tendency to induce systemic hypertension at higher doses in certain species necessitates further research to verify its safety and efficacy across various animal species [29,78]. As of now, no agonist targeting A_3_ARs has been advanced into clinical trials for the treatment of heart disease, highlighting a gap in the translation of preclinical findings to potential therapeutic applications for A_3_AR agonists in cardiovascular medicine.

## 6. Clinical Studies of A_3_AR Agonists for the Treatment of Non-Cardiac Diseases

In addition to its role in cardiovascular diseases, A_3_AR has been highlighted across multiple cell types implicated in various pathological conditions, especially cancer and inflammation [15,51,83]. Consequently, there is substantial interest in exploring its potential as a therapeutic target for both anti-tumor and anti-inflammatory therapies. This section provides a summary of clinical studies on two selective A_3_AR agonists, namodenoson and piclidenoson, detailing research progress and potential applications of A_3_AR agonists in non-cardiac diseases. These include non-alcoholic fatty liver disease (NAFLD), hepatocellular carcinoma (HCC), and chronic plaque psoriasis, as outlined in Table 3.

### 6.1. Namodenoson (2Cl-IB-MECA; CF-102)

NAFLD, characterized by excessive fat accumulation in the liver associated with metabolic dysfunction, ranks among the most prevalent chronic liver disorders globally, impacting approximately 25% of the adult population [84]. NAFLD is considered the most common cause of chronic liver disease such as cirrhosis (liver fibrosis) and HCC [84,85]. Besides NAFLD, HCC is also one of the major global health problems, accounting for about 75–85% of all liver cancer cases [86]. Interestingly, it has been reported that the expression of A_3_ARs is higher in liver cells derived from inflammatory and tumor tissues, but not in normal liver cells [83,87]. Furthermore, targeting A_3_ARs has demonstrated the ability to elicit anti-inflammatory and anti-cancer effects, emphasizing the promising prospect of developing A_3_AR agonists as a treatment option for liver diseases [88].

To assess the efficacy and safety of namodenoson, a phase II randomized, dose-finding trial was performed in NAFLD patients aged older than 18 years with or without non-alcoholic steatohepatitis (NASH) (*N* = 60) [89]. The trial administered two distinct doses of namodenoson (12.5 or 25 mg twice daily) for 12 weeks (Table 3). Patients receiving namodenoson, particularly in the 25 mg group, showed a pronounced decrease in serum ALT levels, an indicator of liver inflammation, compared to the placebo group, with a higher proportion achieving ALT normalization at 16 weeks. Concurrently, improvements in other liver disease-related parameters such as AST and adiponectin were evident, with both doses demonstrating good tolerability [89]. Overall, namodenoson led to improvements in liver function and inflammation, particularly at the higher dose. However, this study was limited by several factors including a small number of patients, a short treatment period, and the absence of post-treatment liver biopsy to monitor the effect of the drug on the liver [89].

HCC and cirrhosis are intricately linked, with cirrhosis serving as a major risk factor for HCC, thereby affecting liver function and mutually influencing each other, presenting a particularly challenging clinical scenario [90]. A phase II randomized clinical trial was conducted to evaluate the efficacy and safety of namodenoson as a second-line therapy (after failing first-line sorafenib) in advanced HCC and Child-Pugh B cirrhosis patients (*N* = 78) [91]. Patients were randomized to receive either namodenoson (25 mg twice daily) or placebo until discontinuation (Table 3). Regrettably, namodenoson did not demonstrate an improvement in overall survival (primary endpoint) compared to placebo (median overall survival, 4.1 months vs. 4.3 months, *p* = 0.46). However, namodenoson was well tolerated without any adverse events, and a significant improvement in 12-month overall survival was documented in a subgroup of patients with a Child-Pugh score of 7 (44% vs. 18%, *p* = 0.028) [91].

**Table 3:** Clinical studies of A_3_AR agonists in non-cardiac diseases.

Drug	Study Population	Treatment	Primary Endpoints	Main Findings
Namodenoson (Phase II) [89]	NAFLD patients with or without NASH (*N* = 60)	Namodenoson (12.5 or 25 mg) or placebo, BID, for 12 weeks	Serum ALT level	▪ Decreased ALT levels in a dose-dependent manner ▪ Improved liver function and inflammation at a dose of 25 mg
Namodenoson (Phase II) [91]	Patients with advanced HCC and Child-Pugh B cirrhosis (*N* = 78)	Namodenoson (25 mg) or placebo, BID	Overall survival	▪ The study did not meet primary endpoint ▪ Demonstrated a favorable safety profile ▪ Improved 12-month overall survival in a subgroup of patients with a Child-Pugh score of 7
Piclidenoson (CF-101) (Phase I) [92]	Healthy men (*N* = 43)	CF-101 (1, 5, or 10 mg) or placebo, single dose and repeated doses of up to 4 mg BID, for 7 days	Safety, tolerability, pharmacokinetics, and hemodynamic profiles	▪ Single doses up to 5 mg was well tolerated ▪ At a dose of 10 mg, flushing, tachycardia, nausea, and vomiting were observed ▪ Increase in heart rate (related to plasma levels), but no evidence of tachycardia and PR prolongation
Piclidenoson (CF-101) (Phase II/III) [93]	Patients with moderate-to-severe chronic plaque psoriasis (*N* = 293)	CF-101 (1 or 2 mg) or placebo, BID, followed up to 32 weeks	Proportion of patients achieving ≥ 75% improvement in PASI	▪ The primary endpoint was achieved in only 2 mg BID of CF-101 ▪ CF-101 was safe and well tolerated
Piclidenoson (Phase III; COMFORT-1) [94]	Patients with moderate-to-severe chronic plaque psoriasis (*N* = 529)	Piclidenoson (2 or 3 mg), apremilast (30 mg), or placebo, BID, for 16 weeks	Proportion of patients achieving ≥ 75% improvement in PASI	▪ The primary endpoint was achieved in only 3 mg BID of piclidenoson ▪ The efficacy of piclidenoson was not comparable to that of apremilast

Abbreviations: ALT, alanine aminotransferase; BID, twice daily; HCC, hepatocellular carcinoma; NAFLD, non-alcoholic fatty liver disease; NASH, non-alcoholic steatohepatitis; PASI, psoriasis area and severity index.

### 6.2. Piclidenoson (IB-MECA; CF-101)

To evaluate the safety, tolerability, and pharmacokinetics of piclidenoson, a phase I clinical study in healthy men in Europe was conducted as shown in Table 3. Following oral administration, piclidenoson reached its maximum concentration (C_max_) of 81.6 ng/mL from a single 5 mg dose, with a peak time (T_max_) of 1–2 h and a half-life (T_1/2_) of about 9 h in healthy men [92]. Single oral doses of up to 5 mg and repeated doses of up to 4 mg every 12 h for 7 days were well tolerated, while higher doses caused side effects like flushing, tachycardia, nausea, and vomiting [92]. However, given that medications are frequently administered intravenously for treating patients with acute myocardial infarction, the safety and pharmacokinetics of piclidenoson should be investigated via this route.

Piclidenoson has been elucidated in the context of psoriasis, a type of autoimmune disease characterized by the rapid proliferation of keratinocytes, resulting in itchy and red patches of skin covered with silvery scales. In phase II/III trials, CF-101 (piclidenoson) demonstrated the safety profile in patients with moderate-to-severe plaque psoriasis [93]. The majority of patients were males (63.5%) and White/Caucasian (99.0%). Recently, the efficacy and safety of piclidenoson were further investigated in psoriasis patients through the COMFORT-1 study, a phase 3 clinical trial (Table 3). This study enrolled 529 patients with moderate-to-severe chronic plaque psoriasis, who were randomized to receive piclidenoson (2 or 3 mg), apremilast (30 mg), or placebo twice daily [94]. Both doses of piclidenoson exhibited comparable efficacy and favorable safety profiles. However, the primary endpoint, the proportion of patients achieving ≥ 75% improvement in psoriasis area and severity index (PASI) from baseline to 16 weeks, was achieved only in 3 mg BID of piclidenoson (9.7% vs. 2.6%, piclidenoson vs. placebo, *p* = 0.037) [94]. Furthermore, a noninferiority test comparing the efficacy of piclidenoson and apremilast revealed that neither the 3 mg nor the 2 mg doses of piclidenoson displayed comparable efficacy to apremilast in improving psoriasis, as assessed with PASI-75 after 32 weeks of treatment (17.0% vs. 20.8% vs. 26.2%, respectively; *p* = 0.88 and *p* = 0.43 for the respective comparisons vs. apremilast). Overall, although the effectiveness of piclidenoson is not comparable to that of apremilast, its beneficial effects show a trend toward improvement, supporting further development of piclidenoson for treating psoriasis [94].

## 7. Limitations and Conclusions

Today, the pharmaceutical landscape is actively engaged in the development of various drug targets for the management of CVDs, particularly heart diseases [95,96], among which A_3_AR stands out as a promising candidate, although it has not yet received approval. In recent years, increasing knowledge about the role of A_3_ARs in heart diseases has been documented in several preclinical models. Even though some findings suggest that modulating A_3_AR signaling confers a cardioprotective function, the available preclinical and clinical data remain insufficient for application in patient care. The dynamic expression of A_3_ARs across different tissue contexts in pathological states, the genetic variations of A_3_AR signaling, and disparities in agonist/antagonist binding profiles between rodents and humans underscored the limitations in directly extrapolating findings from animal models to humans. Moreover, the specificity of A_3_AR-selective ligands may have been compromised, particularly at higher concentrations, leading to unintended off-target effects on other AR subtypes. Furthermore, experimental techniques for studying A_3_AR function are limited, requiring new pharmacological probes and molecular methods to validate its expression in mammalian cardiomyocytes. Gene-knockout studies have provided insights into the functional roles of A_3_ARs but may trigger compensatory mechanisms that influence experimental outcomes. It was noteworthy that while A_3_AR activation demonstrates positive effects, excessive A_3_AR activation might have contributed to pathological cardiac remodeling, including hypertrophy and fibrosis. Lastly, a significant limitation of A_3_AR agonists and antagonists was their potential adverse effects, which must be carefully considered.

In conclusion, our current review offered updated insights into the signal transduction pathways of A_3_ARs. These pathways notably involved G protein-dependent signaling cascades, particularly involving G_i_ and G_q_ proteins, alongside several downstream signaling effectors. Today, various A_3_AR agonists have shown protective effects and demonstrated efficacy in preventing arrhythmias, protecting against myocardial infarction, and reducing cardiac hypertrophy, fibrosis, and myocardial dysfunction in preclinical models of heart diseases. However, the role of A_3_AR antagonists in these contexts remains less explored, with limited available data. While existing A_3_AR agonists or antagonists are still in early-stage clinical trials, their potential therapeutic implications for heart diseases remain largely unexplored. Nevertheless, animal studies have provided promising insights, bringing the therapeutic application of A_3_AR-targeting compounds notably closer to realization.

## Data Availability

All data generated or analyzed during the current study are included in this published article.

## Abbreviations

BID: twice daily; CCPA: 2-chloro-N^6^-cyclopentyladenosine; CPA: N^6^-cyclopentyl-adenosine; DAG: diacylglycerol; EGR: growth response factors; ERK: extracellular signal-regulated kinase; GSK-3β: glycogen synthase kinase-3 beta; HR: heart rate; IP_3_: inositol triphosphate; I/R: ischemia-reperfusion; MEK1: mitogen-activated protein kinase kinase 1; mitoK_ATP_ channel: mitochondrial ATP-sensitive potassium channel; PI3K: phosphoinositide 3-kinase; PKC: protein kinase C; PLC: phospholipase C; PLD: phospholipase D; RhoA: Ras homolog family member A; PASI: psoriasis area severity index.
